# Influence of Polyphosphates on the Physicochemical Properties of Poly (Vinyl Chloride) after Irradiation with Ultraviolet Light

**DOI:** 10.3390/polym12010193

**Published:** 2020-01-10

**Authors:** Gamal A. El-Hiti, Dina S. Ahmed, Emad Yousif, Mohammad Hayal Alotaibi, Hind A. Satar, Ahmed A. Ahmed

**Affiliations:** 1 Cornea Research Chair, Department of Optometry, College of Applied Medical Sciences, King Saud University, P.O. Box 10219, Riyadh 11433, Saudi Arabia; 2 Department of Medical Instrumentation Engineering, Al-Mansour University College, Baghdad 64021, Iraq; dinasaadi86@gmail.com; 3 Department of Chemistry, College of Science, Al-Nahrain University, Baghdad 64021, Iraq; hind10111993@gmail.com (H.A.S.); ahmedahmedalazawi@gmail.com (A.A.A.); 4 National Center for Petrochemicals Technology, King Abdulaziz City for Science and Technology, P.O. Box 6086, Riyadh 11442, Saudi Arabia

**Keywords:** poly(vinyl chloride), polyphosphates, photostabilization, depression in average molecular weight, indices of functional groups, surface morphology

## Abstract

Three new polyphosphates were synthesized in good yields by reacting diethylenetriamine with the appropriate phosphate ester in ethanol under acidic conditions. The polyphosphate structures were determined using FT-IR and ^1^H-NMR spectroscopies, and their elemental compositions were confirmed by EDX spectroscopy. Polyphosphates were added to poly(vinyl chloride) (PVC) at low concentrations to fabricate thin films. The PVC films were irradiated with ultraviolet light for long periods, and the effect of polyphosphates as the photostabilizer was investigated by determining changes in the infrared spectra (intensity of specific functional group peaks), reduction in molecular weight, weight loss, and surface morphology. Minimal changes were seen for PVC films containing polyphosphate compared to that for the blank film. In addition, optical, scanning electron, and atomic force microscopies were used to inspect the surface morphology of films. Undesirable changes due to photodegradation were negligible in PVC films containing additives compared to films containing no additives. In addition, the surfaces were smoother and more homogeneous. Polyphosphates, and in particular ones that contain an *ortho*-geometry, act as efficient photostabilizers to reduce the rate of photodegradation. Polyphosphates absorb ultraviolet light, chelate with polymeric chains, scavenge radical moieties, and decompose peroxide residues.

## 1. Introduction

Plastics are used for everyday applications that range from food packaging to medical tools. Poly(vinyl chloride) (PVC) is a common thermoplastic and commercially valuable. A large proportion (more than 50%) of the produced PVC is used in construction materials since it can be assembled easily and is inexpensive to produce [1]. The mechanical, physical, and chemical properties of PVC can be modulated to produce different degraded polymeric materials that vary in molecular structures and forms [2]. The common forms of PVC are flexible and rigid PVC [3]. Rigid PVC is used in the fabrication of windows, doors, bottles, cards, and food packaging. PVC can be produced in a soft form (flexible chains) when a plasticizer (e.g., phthalates) is added [4]. Soft PVC can be used in insulators, flooring, and palming (i.e., a rubber replacement). For use in outdoor building applications, PVC should be stable enough to resist changes from exposure to sunlight over long durations. Currently, PVC suffers from poor light resistance and thermal stability when exposed to ultraviolet (UV) irradiation. Photodegradation of PVC leads to undesirable changes within the polymeric material due to bond scissions that lead to unwanted chemical transformations [5,6]. As a result, PVC loses some of its mechanical, electrical, and optical properties that result in discoloration, crack formation, erosion, cross-linking, and transparency loss [7]. PVC dehydrochlorination occurs at high temperatures, in which hydrogen chloride (HCl) and double bond fragments (e.g., conjugated polyenes) are produced, decreasing the molecular weight of the polymer [8,9,10]. To overcome the problems associated with PVC photodegradation, additives are used to enhance its stability against irradiation [11,12].

Various commercial additives have been added to PVC to act as a light absorber, heat stabilizer, peroxide decomposer, excited state quencher, radical scavenger, smoke suppressor, or flame retardant [4]. Such additives must be compatible with the PVC material, non-volatile, non-hazardous, should not cause discoloration, and is cheap to produce. For example, 3,3′,4,4′-tetrachlorobiphenyl, *bis*(2-ethylhexyl)phthalate, *tris*(di-*tert*-butylphenyl)phosphite, and barium-zinc stabilizer have been used as PVC additives on a commercial scale [13,14]. However, some of these additives are carcinogenic or require the use of co-stabilizers [15,16]. Therefore, new additives are currently being researched and developed to stabilize PVC. Recently, several additives have been synthesized and tested as PVC photostabilizers to inhibit its photodegradation. For example, Schiff bases [17,18,19,20,21,22,23], highly aromatic compounds [24,25,26,27], porous polyphosphates [28,29,30], organotin complexes [31,32,33,34], titanium dioxide [35,36], and others [37,38,39] can significantly reduce the PVC photodegradation rate upon exposure to UV irradiation for long periods of time. Herein, we report the facile synthesis of three new polyphosphates derived from diethylenediamine and investigate their ability to protect PVC films against UV irradiation [40,41,42,43,44,45]. Indeed, the synthesized polyphosphates significantly reduced PVC photodegradation when added to the polymeric materials at low concentrations.

## 2. Materials and Methods

### 2.1. Instrumentation

PVC (*M* = ca. 250,000; *K* value = 67, polymerization degree = 800) was obtained from Petrokimya (Istanbul, Turkey). The FT-IR spectra were recorded on Shimadzu 8300 Spectrophotometer (400–4000 cm^−1^) using the potassium bromide disk method. ^1^H-NMR spectra (500 MHz) were recorded on Bruker DRX500 NMR Spectrometer (Bruker, Zürich, Switzerland) using tetramethylsilane as the internal standard in deuterated dimethyl sulfoxide. A Kerry PUL 55 ultrasonic bath (Kerry Ultrasonics Ltd., Hitchin, UK) was used for the preparation of the PVC blends. Aluminum plates (thickness = 0.6 mm) obtained from Q-Panel Company (Homestead, FL, USA) were used to fix the PVC films. The thickness of the PVC films (approximately 40 μm) was measured using a Digital Caliper DIN 862 micrometer (Vogel GmbH, Kevelaer, Germany). PVC films were irradiated at 365 nm using an accelerated weather-meter QUV tester obtained from Q-Panel Company (Homestead, FL, USA) with a UV light intensity of 6.43 × 10^−9^ ein dm^−3^·s^−1^ at 25 °C. The viscosity of PVC was measured using an Ostwald U-Tube Viscometer (Ambala, Haryana, India). Energy dispersive X-ray (EDX) images were recorded on a Bruker XFlash 6 10 (Bruker, Tokyo, Japan). Microscopic images of the PVC surface were recorded on a Meiji Techno Microscope (Tokyo, Japan). The scanning electron microscopy (SEM) images were captured at an accelerating voltage of 15 kV using an Inspect S50 microscope (FEI Company, Czechia, Czech Republic). Atomic force microscopy (AFM) images were recorded on a Veeco instrument (Veeco Instruments Inc., Plainview, NY, USA).

### 2.2. Synthesis of Polyphosphates **1**–**3**

A mixture of appropriate *tris*(formylphenyl)phosphate (8.21 g, 20 mmol) and diethylenetriamine (3.24 g, 30 mmol) in boiling dry EtOH (25 mL) containing glacial ACO_2_H (0.5 mL) was stirred in a round-bottomed flask (100 mL) for 6 h. The solid formed upon cooling was filtered, washed with EtOH (3 × 10 mL), and dried in a vacuum oven to afford polyphosphates **1**–**3**. *tris*(Formylphenyl)phosphates were synthesized as reported from reacting appropriate hydroxybenzaldehyde and phosphoryl chloride in the presence of triethylamine in tetrahydrofuran (THF) [41].

### 2.3. Preparation of PVC Films

A mixture of PVC (5 g) and polyphosphates **1**–**3** (25 mg) in THF (100 mL) was stirred for 30 min at 25 °C The homogeneous mixture was transferred to clean glass plates with a thickness of approximately 40 μm. The films produced were dried for 36 h at 25 °C and for 12 h in a vacuum oven.

## 3. Results

### 3.1. Characterization of Polyphosphates **1**–**3**

**Figure 1:**
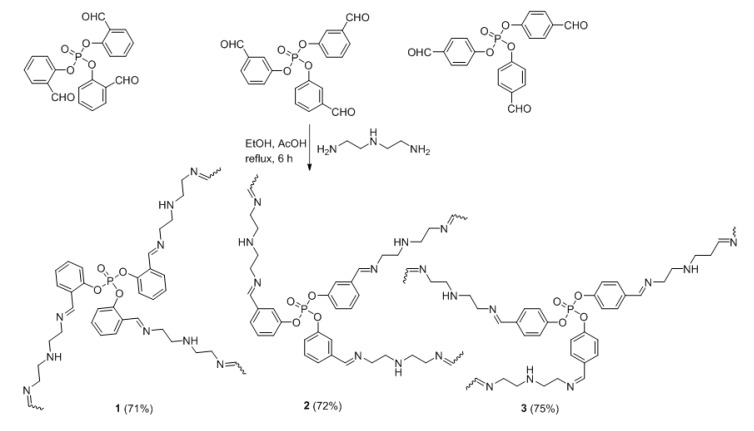
Polyphosphates **1**–**3**.

Reactions of *tris*(formylphenyl)phosphates, obtained from reactions of phosphoryl chloride and *ortho*-hydroxy, *meta*-hydroxy, and *para*-hydroxybenzaldehydes in a basic medium [41], and excess diethylenetriamine (three molar equivalents) in dry ethanol (EtOH) containing acetic acid (AcO_2_H) for 6 h under reflux gave polyphosphates **1**–**3**. The resulting polyphosphates **1**–**3** (Figure 1) were obtained as orange solids in good yields (Table 1).

The structures of polyphosphates **1**–**3** were confirmed by FT-IR and ^1^H-NMR. The FT-IR spectra of **1**–**3** showed the presence of absorption bands corresponding to the P=O, P–O–C, C=C, and CH=N groups that appeared at 1165–1188, 1222–1242, 1556–1585, and 1631–1643 cm^−1^, respectively (Table 2). Moreover, the stretching bands that correspond to the carbonyl and amino groups of substituted benzaldehydes and diethylenediamine, respectively, were absent in the FT-IR spectra of **1**–**3**. The ^1^H-NMR spectra of **1**–**3** showed singlets that appeared at 8.51–8.24 ppm, corresponding to the azomethine (CH=N) protons (Table 3). The aromatic and CH_2_CH_2_ protons appeared as multiplets at 7.68–7.22 and 3.48–2.64 ppm, respectively.

### 3.2. The EDX Spectroscopy of PVC Films

**Figure 2:**
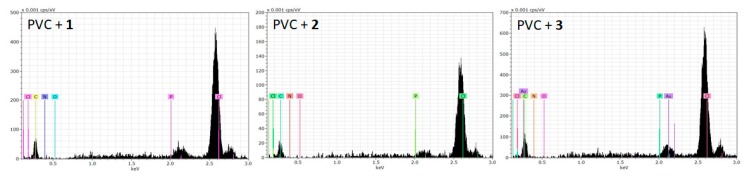
Energy dispersive X-ray (EDX) patterns of PVC films.

Polyphosphates **1**–**3** were mixed with PVC at a concentration of 0.5% by weight, based on previous reports [24], to prepare polymeric films (thickness = 40 mm). The use of low concentrations leads to homogeneity without film discoloration. EDX spectroscopy was used to identify the elements within the polymer films [46]. The EDX patterns of the PVC blends containing additives **1**–**3** showed strong absorption bands, corresponding to the elements in both PVC and polyphosphates (Figure 2). The assignment of bands in Figure 2 agree with assignments reported elsewhere in the literature [23,33].

**Figure 3:**
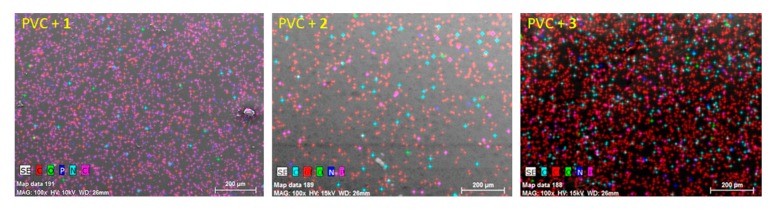
EDX chemical mapping patterns of PVC films.

The EDX chemical mapping analysis of the PVC/polyphosphates blends is shown in Figure 3. The mapping images revealed a remarkable point density of elements from both PVC and the polyphosphates (carbon, chlorine, nitrogen, oxygen, and phosphorous), which are homogeneously distributed within the surface of the polymeric blends. The surface atomic percentage of the elements within the PVC films was similar, but is difficult to be precise.

### 3.3. Fourier-Transform Infrared (FT-IR) Spectroscopy of PVC Films

**Figure 4:**
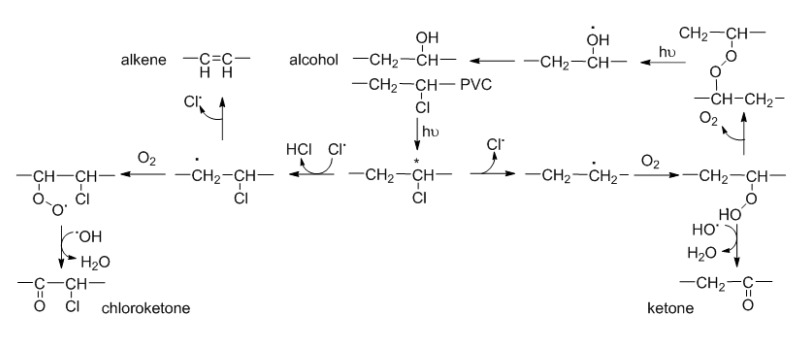
Formation of polyene, carbonyl, and hydroxyl containing fragments from PVC photo-oxidation.

When PVC is exposed to UV irradiation for long durations in the presence of oxygen, a photo-oxidative degradation process occurs, which leads to the formation of free radical species, causing cross-linking of the polymeric chains and undesirable changes in electrical, optical, mechanical, and chemical properties of the polymeric materials. In addition, PVC photo-oxidation results in the production of small polymeric fragments that contain carbonyl groups, such as acid chloride, chlorocarboxylic acid, chloroketones, and ketones (Figure 4) [47]. Small fragments containing hydroxyl and polyene groups are also produced (Figure 4).

**Figure 5:**
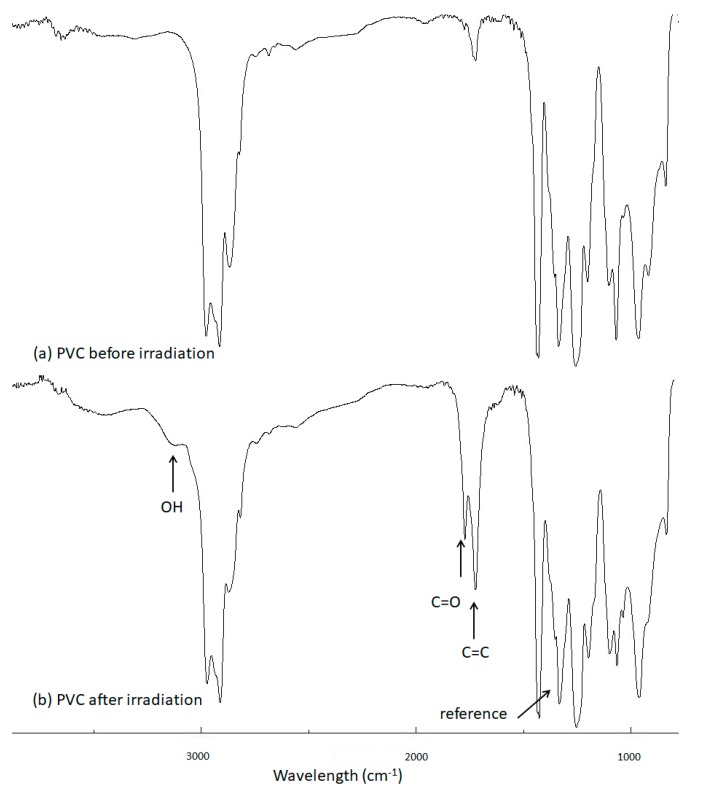
FT-IR spectra of PVC (**a**) before and (**b**) after irradiation.

The FT-IR spectra of PVC films (40 µm in thickness) irradiated for 300 h using UV light (λ_max_ = 313 nm) were recorded and compared to those obtained before irradiation. The FT-IR spectra of PVC in the absence of the polyphosphate additives before and after irradiation are shown in Figure 5. The FT-IR spectra shows that the intensity of the peaks at 1602, 1722, and 3500 cm^−1^, corresponding to the vibrations of C=C (polyene), C=O (carbonyl), and OH (hydroxyl) moieties, respectively, increased during irradiation, with the highest intensity exhibited after 300 h (Figure 5).

PVC films in the absence and presence of **1**–**3** were irradiated with UV light for 300 h, and the FT-IR spectra were recorded at 50 h intervals. The intensities of peaks for the C=C (1602 cm^−1^), C=O (1722 cm^−1^), and OH (3500 cm^−1^) vibrations were compared to a reference peak (1328 cm^−1^, C–H bond vibrations). The indices (*Is*) of the functional groups were then calculated from the functional group (*As*) and reference group (*Ar*) absorbance using Equation (1) [17].

(1) $$I_{s}\;=\;A_{s}/A_{r}$$

**Figure 6:**
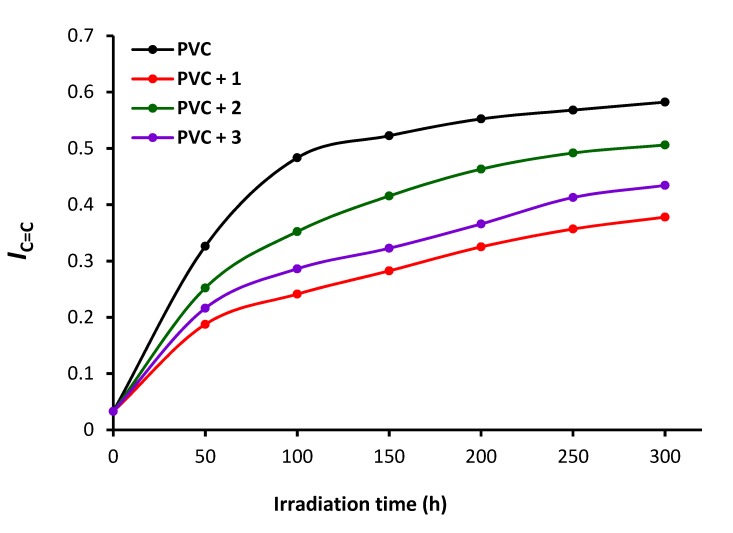
Changes in the *I*C=C upon irradiation.

**Figure 7:**
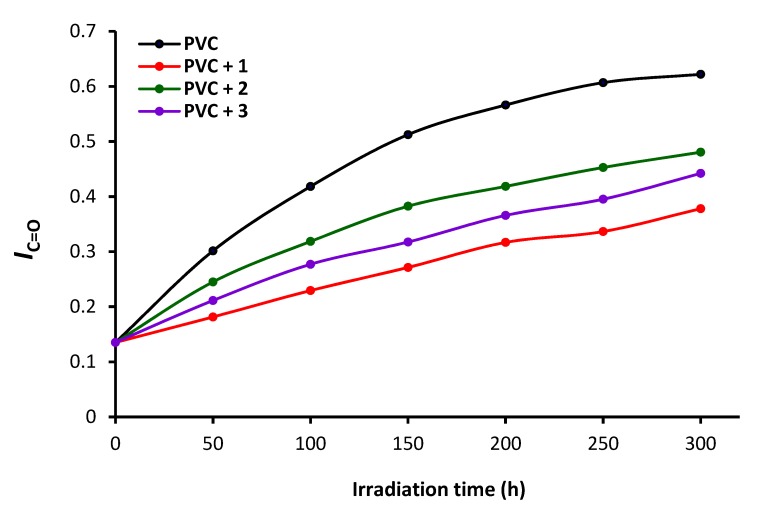
Changes in the *I*C=O upon irradiation.

**Figure 8:**
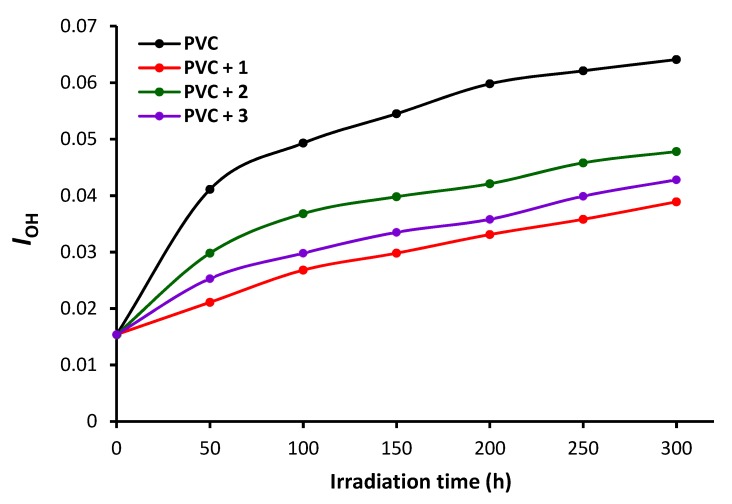
Changes in the *I*OH upon irradiation.

The polyene (*I*C=C), carbonyl (*I*C=O), and hydroxyl (*I*OH) indices of the blank and polyphosphate-containing PVC films were calculated at different irradiation times (Figure 6, Figure 7 and Figure 8). The irradiation of each PVC film and monitoring of the changes in these functional group indices was carried out for only one time. Greater changes in the functional indices were seen for the blank PVC films compared to those for films containing polyphosphates **1**–**3** as additives. These results confirm that the polyphosphate additives act as efficient PVC photostabilizers. The lowest change in indices was achieved when polyphosphate **1** (*ortho*-geometry) was added. The efficiency of additives as PVC photostabilizers are: **1** (*ortho*-geometry) > **3** (*meta*-geometry) > **2** (*para*-geometry).

### 3.4. Weight Loss (%) of PVC Films

Dehydrochlorination of PVC takes place at high temperatures and leads to a loss in polymeric material due to the elimination of hydrogen chloride [48]. Such a process also leads to the evolution of volatile toxic pollutants and discoloration of the polymeric material. The use of polyphosphates **1**–**3** can stabilize the PVC against long-term UV irradiation. The PVC films were irradiated for a long period of time, and the weight loss (%) was calculated every 50 h using Equation (2) [17], wherein *W*_1_ is the PVC film weight before irradiation and *W*_2_ is the PVC film weight at the appropriate time point.

(2) $$Weight\;loss\;\%\;=\;\left[\left(W_{1}-W_{2}\right)/W_{1}\right]\;\times \;100$$

**Figure 9:**
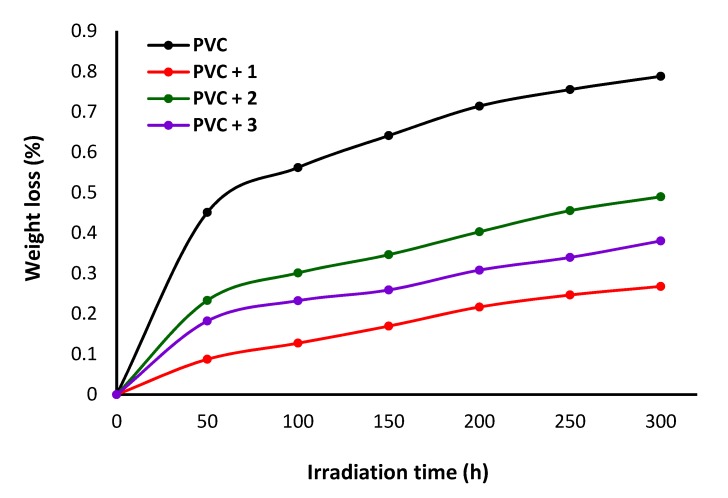
Changes in weight loss (%) upon irradiation.

The changes in weight loss (%) of the PVC films at different UV irradiation time points are shown in Figure 9. The weight loss from the blank PVC film was higher than those obtained from the films containing polyphosphates. The lowest loss in weight was demonstrated by the PVC film with polyphosphate **1**. The efficiency of polyphosphates as PVC photostabilizers are: **1** > **3** > **2**.

### 3.5. Viscosity-Average Molecular Weight (${\bar{M}}_{V}$) of PVC Films

**Figure 10:**
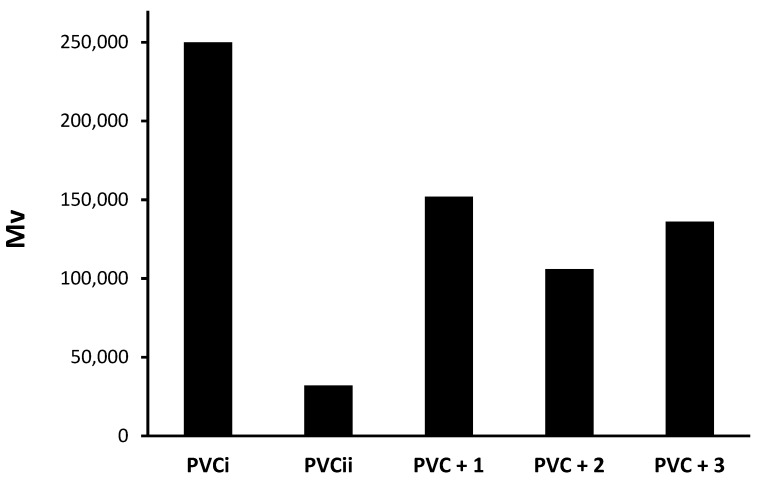
Molecular weight (${\bar{M}}_{V})$ for PVC films decreases after irradiation. PVCi and PVCii represent the ${\bar{M}}_{V}$ of PVC before and after irradiation (300 h), respectively.

In solution, the viscosity-average molecular weight (${\bar{M}}_{V}$) of PVC is directly proportional to its intrinsic viscosity ([η]). The PVC films were irradiated for 300 h, dissolved in anhydrous THF, and their viscosities were measured using an Ostwald U-Tube viscometer [49]. Equation (3) was used to calculate the ${\bar{M}}_{V}$ (g/mol) of PVC after irradiation [50,51]. For comparison, the ${\bar{M}}_{V}$ of the non-irradiated PVC (blank) film was calculated. Figure 10 displays the decrease in the ${\bar{M}}_{V}$ for irradiated PVC films after 300 h compared to the case for non-irradiated films. The results shown in Figure 10 are based on the irradiation of PVC films for only one time. A substantial decrease in ${\bar{M}}_{V}$ was demonstrated for blank PVC (87%), as it was reduced from 250,000 to 32,000 after 300 h of irradiation. The reduction in the ${\bar{M}}_{V}$ for PVC films containing additives **1**–**3** ranged from 39% to 58%, in which polyphosphate **1** was the most effective additive in stabilizing the PVC film. In the presence of **1**, the ${\bar{M}}_{V}$ was reduced from 250,000 to 152,000 after 300 h.

(3) $$\left[\eta \right]=K{\bar{M}}_{V}^{\alpha }$$

### 3.6. Optical Microscopy of PVC Films

**Figure 11:**
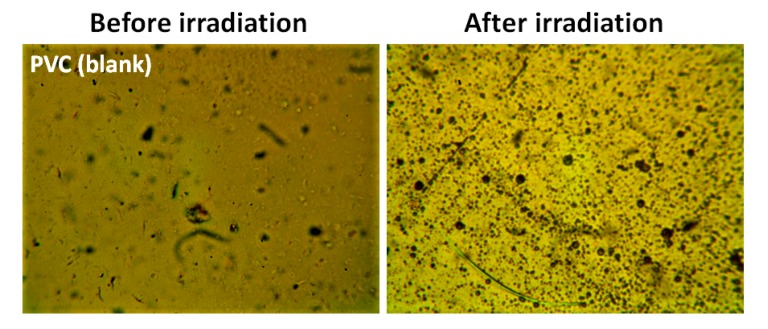
Microscopic images of PVC (blank) film (400× magnification).

**Figure 12:**
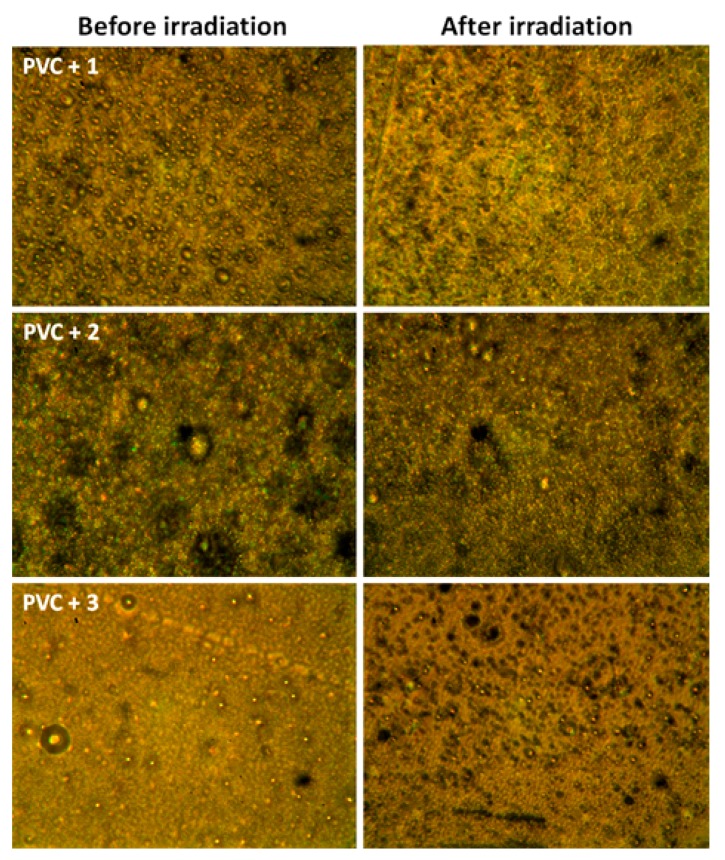
Microscopic images of PVC films containing polyphosphates **1**–**3** (400× magnification).

The polymer surface morphology provides valuable data regarding physical properties, such as crystallinity, surface irregularities, damage, cracks, chain scission, and other defects. Therefore, the surface morphology of the PVC films that were irradiated for 300 h was inspected by optical microscopy. Micrographs were recorded at 400× magnification and are shown in Figure 11. Various reports demonstrate that the surface morphology of PVC before irradiation is smooth and homogenous without cracks or white spots [20,34]. The surface of the irradiated PVC films was heterogeneous, rough, and contained relatively larger numbers of cracks, grooves, holes, and spots compared to the surface of non-irradiated PVC films (Figure 11 and Figure 12). However, the surface irregularities were more pronounced for the blank PVC film (Figure 11) compared to those for films containing polyphosphates **1**–**3** (Figure 12). Clearly, polyphosphates **1**–**3** act as effective PVC stabilizers, as they inhibit the elimination of hydrogen chloride from the polymeric chains. The surface of the PVC film containing **1** was relatively smooth and contained minimal cracks, groves, and white spots, supporting its ability to function as an effective additive to stabilize PVC.

### 3.7. SEM of PVC Films

**Figure 13:**
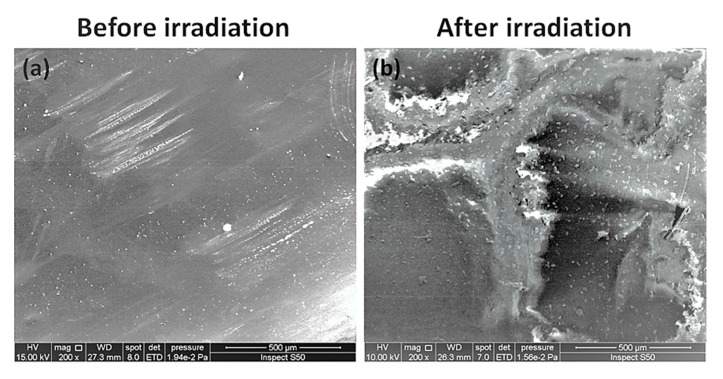
SEM images of the blank PVC film (**a**) before and (**b**) after irradiation.

**Figure 14:**
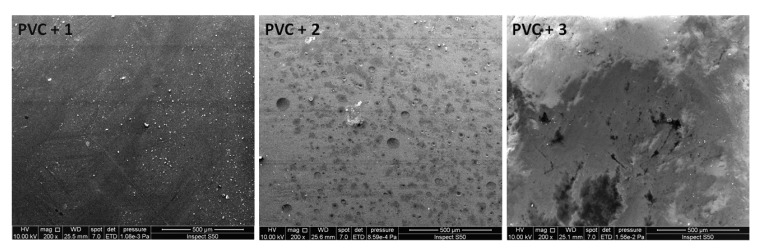
SEM images of PVC films containing polyphosphates **1**–**3** after irradiation.

The surface morphology of PVC after irradiation was investigated using SEM to provide insight regarding the changes that occur within the surface of PVC films. In addition, SEM was used to estimate the PVC particle shape and size, its ionic conductivity, and thermal and mechanical stability [52]. Various reports indicate that the surface of non-irradiated PVC films is smother and more homogenous compared to that irradiated with UV light [32,34]. Figure 13 shows that irradiating PVC films for a long period of time in the absence of additives leads to a high degree of irregularities, roughness, and defects within the polymeric surface. However, Figure 14 shows that such defects and irregularities are less pronounced in the presence of polyphosphates compared to the case of the blank film. The surface irregularities are due to polymeric chain cross-linking and the formation of volatile residues, such as hydrogen chloride [52].

### 3.8. AFM of PVC Films

**Figure 15:**
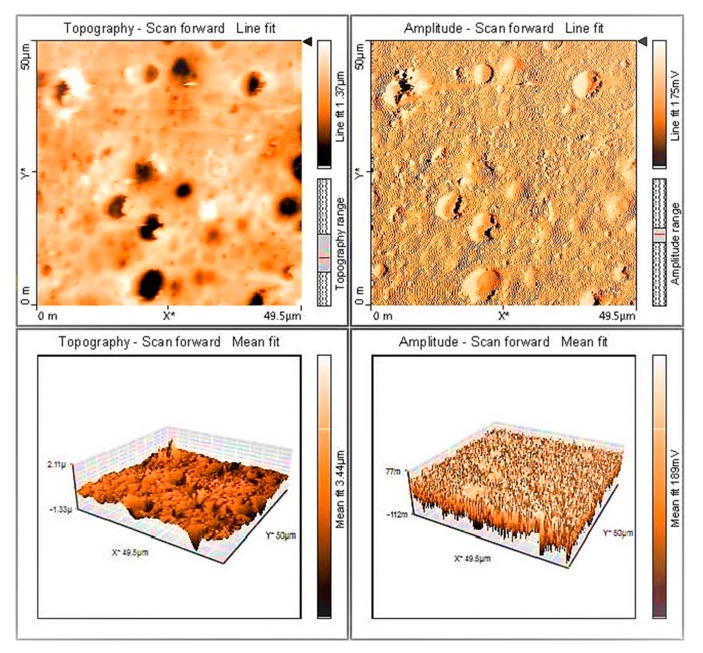
Two-dimensional (2D) and 3D AFM images of PVC containing polyphosphate **1** after irradiation.

Various reports suggest that AFM can be used to investigate the irregular and rough polymer surface of materials exposed to irradiation [23,53,54]. PVC films containing polyphosphate **1** were irradiated for 300 h, after which, 2D and 3D AFM images of the surface were captured. Figure 15 shows a relatively regular packaging of lamellar crystals that have different sizes and shapes. In order to confirm that polyphosphates act as PVC photostabilizers, the roughness factor (*Rq*) was measured for the irradiated PVC films. The *Rq* for irradiated blank PVC, PVC + **1**, PVC + **2**, and PVC + **3** films were 390.1, 46.3, 86.9, and 82.3, respectively. Clearly, polyphosphate **1** produced the greatest photostabilizing effect and improved the roughness factor by more than 9-fold compared to the blank PVC film. This result indicates that the rate of both dehydrochlorination and bond-breaking was significantly reduced in the presence of **1** [55,56].

Table 4 shows the fold improvement in *Rq* for PVC films using different additives. Clearly, polyphosphates, which contain a high degree of aromaticity, lead to the greatest improvement in *Rq* [28]. Aromatic residues stabilize PVC against irradiation through direct absorption of UV light. Some organotin complexes are also effective in stabilizing PVC films against irradiation [32].

### 3.9. Photostabilization of PVC Mechanism

**Figure 16:**
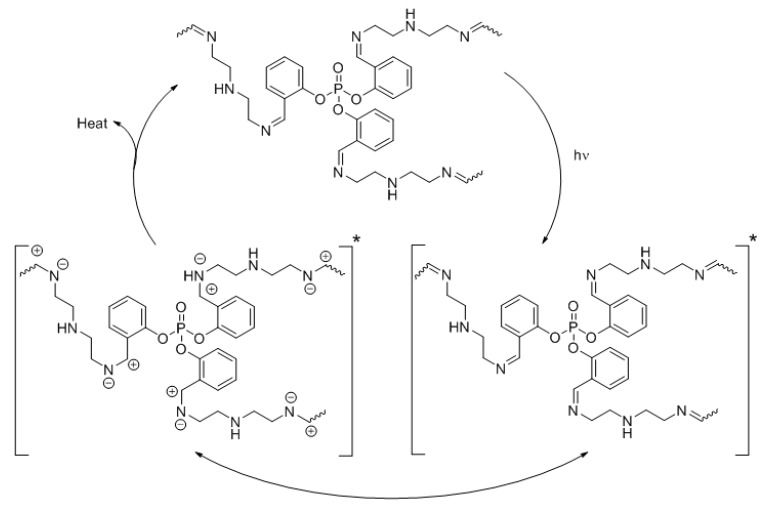
Polyphosphate **1** acts a UV absorber.

Irradiation of PVC damages the polymeric materials due to the formation of excited electrons [57,58]. Polyphosphates **1**–**3**, containing aromatic moieties, act as UV absorbers [25,26] and release energy as heat at harmless levels for the PVC chains (Figure 16).

**Figure 17:**
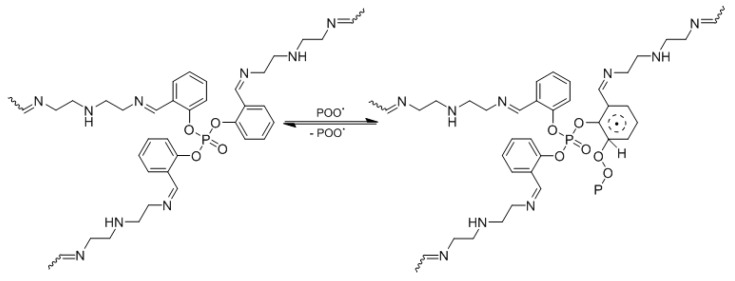
Polyphosphate **1** acts as a radical scavenger.

Polyphosphates **1**–**3** act acts as radical scavengers when a chromophore (POO^•^) is present [59]. For example, polyphosphate **1** produces a stable complex with the chromophore, allowing the energy to be transferred as a result of resonating aryl rings, which stabilizes the PVC films when exposed to irradiation (Figure 17).

**Figure 18:**
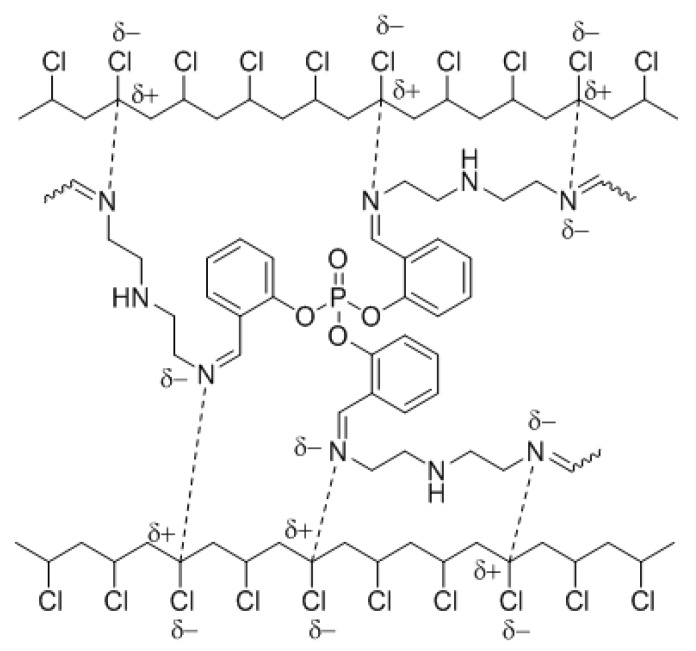
Interaction between polyphosphate **1** and PVC chains.

The coordination between the polarized nitrogen of the azomethine moieties in polyphosphates and the polarized carbon of the C–Cl bonds in the PVC chains stabilizes the polymeric materials. Such interactions lead to a transfer of the PVC excited state energy to the polyphosphates (Figure 18). The higher efficiency of polyphosphate **1** as a PVC photostabilizer compared to those of **2** and **3** is due to the effective absorption of the UV light as a result of the *ortho*-arrangement.

## 4. Conclusions

New polyphosphates containing diethylenetriamine and aromatic moieties were synthesized in good yields, characterized, and tested as potential photostabilizers for PVC against UV irradiation over long durations. Polyphosphates were added at a low concentration to avoid film discoloration and maintain homogeneity. PVC films were UV irradiated for 300 h, and the changes on the surface or within the polymer were monitored and analyzed. Undesirable changes to PVC, such as discoloration, the formation of functional groups, reduction in molecular weight, weight loss, cross-linking, and irregularities within the surface were less noticeable when polyphosphates were used as additives compared to the case of the blank film. Clearly, polyphosphates, particularly with an ortho-geometry, act as peroxide decomposers, radical scavengers, and ultraviolet absorbers.

## Figures and Tables

**Table 1:** Color, melting points, and yields (%) of **1**–**3**.

Polyphosphate	Color	Melting Point (°C)	Yield (%)
**1**	Deep orange	155–157	71
**2**	Orange	168–170	72
**3**	Light orange	162–165	75

**Table 2:** FT-IR spectral data for **1–3**.

Polyphosphate	FT-IR (Wavenumber, cm^–1^)			
	P=O	P-O-C	C=C	CH=N
**1**	1188	1222	1581	1631
**2**	1172	1242	1556	1643
**3**	1165	1242	1585	1643

**Table 3:** ^1^H-NMR spectral data for **1**–**3**.

Polyphosphate	^1^H-NMR (Chemical Shift, Ppm)
**1**	8.51 (s, 3H, CH), 7.68–7.25 (m, 12H, Ar), 5.41 (s, exch., 3H, NH), 3.31–3.21 (m, 12H, CH_2_CH_2_), 2.88–2.74 (m, 12H, CH_2_CH_2_)
**2**	24 (s, 3H, CH), 7.45–7.22 (m, 12H, Ar), 5.34 (s, exch., 3H, NH), 3.43–3.23 (m, 12H, CH_2_CH_2_), 2.78–2.67 (m, 12H, CH_2_CH_2_)
**3**	8.45 (s, 3H, CH), 7.60 (d, *J* = 8.6 Hz, 6H, H3/H5 of Ar), 7.22 (d, *J* = 8.6 Hz, 6H, H2/H6 of Ar), 5.22 (s, exch., 3H, NH), 3.48–3.40 (m, 12H, CH_2_CH_2_), 2.72–2.64 (m, 12H, CH_2_CH_2_)

**Table 4:** Improvement roughness factor (*Rq*, fold) of PVC films containing various additives.

PVC Additive	Improvement in *Rq* (Fold)	Reference
Polyphosphates **1**–**3** containing diethylenetriamine	8.4	Current work
Polyphosphate containing benzidine	16.7	[28]
Organotin (IV) complexes	5.2–16.6	[31,32,33,39]
Schiff bases	3.3–6.0	[19,20,23]
